# Social Complexification and Pig (*Sus scrofa*) Husbandry in Ancient China: A Combined Geometric Morphometric and Isotopic Approach

**DOI:** 10.1371/journal.pone.0158523

**Published:** 2016-07-06

**Authors:** Thomas Cucchi, Lingling Dai, Marie Balasse, Chunqing Zhao, Jiangtao Gao, Yaowu Hu, Jing Yuan, Jean-Denis Vigne

**Affiliations:** 1 CNRS, Muséum national d’Histoire naturelle, Sorbonne Universités, UMR 7209, Archéozoologie, Archéobotanique: Sociétés, Pratiques et Environnements, Paris, France; 2 Department of Archaeology, University of Aberdeen, St Mary's, Aberdeen, United Kingdom; 3 History Faculty, Anhui University, Hefei, China; 4 Key lab of vertebrate Evolution and Human Origins of Chinese Academy of Sciences, Institute of Vertebrate Palaeontology and Palaeoanthropology, Chinese Academy of Science, Beijing, China; 5 Institute of Archaeology, Chinese Academy of Science, Beijing, China; New York State Museum, UNITED STATES

## Abstract

Pigs have played a major role in the economic, social and symbolic systems of China since the Early Neolithic more than 8,000 years ago. However, the interaction between the history of pig domestication and transformations in Chinese society since then, have not been fully explored. In this paper, we investigated the co-evolution from the earliest farming communities through to the new political and economic models of state-like societies, up to the Chinese Empire, using 5,000 years of archaeological records from the Xiawanggang (XWG) and Xinzhai (XZ) sites (Henan Province). To trace the changes of pig populations against husbandry practices, we combined the geometric morphometric analysis of dental traits with a study of the stable carbon and nitrogen isotope ratios from bone collagen. The domestication process intensified during the Neolithic Yangshao, prompted by greater selective pressure and/or better herd control against wild introgression. After that, pig farming, in XWG, relied on local livestock and a gradual change of husbandry practices overtime. This was characterized by a gentle increase in millet foddering and animal protein intake, until a complete change over to household management during the Han dynasty. The only rupture in this steady trend of husbandry occurred during the Longshan period, with the appearance of small sized and idiosyncratic pigs with specific feeding practices (relying on millet and household scraps). From three exploratory hypothesis, we explored the possibility of anti-elite pig production in XWG during the Longshan period, as a means to resist incorporation into a new economic model promoting intensified domestic production. This exploratory hypothesis is the most suitable to our dataset; however, numerous areas need to be explored further in order to adequately document the role of pigs in the rise of China’s complex societies.

## Introduction

Understanding how new political and economic models affected indigenous cultures and societies is one of the major challenges in archaeology, mainly tackled through changes in material cultures [1]. Using the animal remains from archaeological sites as a proxy of cultural changes is an alternative approach; which considers that animal husbandry and consumption are both highly culturally driven [2] and central within complex social environments, where their value is both economic and social [3,4]. Such an approach relies on the demographic and morphological changes in domestic animals [5,6], and it has proven to be highly efficient in documenting their role in the social complexification in Middle Chalcolithic Anatolia [3] and during the Romanization of Britain [7], Gaul [8] and Iberia [9].

In Chinese culture and economy, pigs have been the main domestic animal since their earliest domestication process 8,000 years ago [10,11]. Since then, pork consumption has been at the core of all forms of commemoration and festivity. Pig sacrifice is documented from as early as the 6^th^ millennium BC in north-eastern China [12], testifying to their importance in economic, social and symbolic systems [13–15]. This centrality is encapsulated in the modern mandarin character of “family” or “home”

*(家*, *jia)* represented by a pig inside a house, discovered for the first time on a bronze vessel of the Shang culture. China’s current pig production and consumption is symbolic of its economic growth: in the last six years the country produced and consumed almost half of all the world’s pigs [16]. However, despite the predominance of pigs in all aspects of Chinese culture and economy since the Early Neolithic, the role that their husbandry played in the social complexification of China, since then, is poorly documented and understood [4,17].

Social complexification in Chinese history is rooted in the Late Neolithic cultures of the 4^th^ and early 3^rd^ millennium BC. During this time farming communities experienced a drastic population increase, and large villages (up to 40 ha) began to exhibit hierarchic systems in mortuary treatments (i.e. the occurrence of larger graves furnished with prestigious artefacts). On the back of these Late Neolithic changes emerged the rise of regional states, starting with the Longshan culture from the 3^rd^ millennium BC. During this period, villages reached up to 300 ha and were densely clustered. The Longshan period also provides us with the first evidence of large scale fortifications with rammed wall sites [18], and the hierarchy associated with prestigious buildings and specialized production activities [18,19]; suggesting the pooling of resources and manpower in central nodes of local authorities [20]. Social hierarchy in cemeteries is also increasingly visible, with clear socio-economic gaps amongst mortuary treatments. Some scholars have suggested that the climatic deterioration in 2000 BC, at the end of the Longshan period, induced the collapse of these Late Neolithic sites, and promoted the rise of the Erlitou culture and the beginning of the Chinese state during the 2^nd^ millennium BC [21]. However, this climatic impact seems to have been restricted to the south-eastern coast of China. Furthermore, during the Erlitou, despite the unprecedented scale in palatial buildings, craft specialization and elite supervised production, the scale of the towns appears to have been receding, no fortifications have been discovered and cemeteries lack segregated elite burials [20]. The next trajectory pulse towards the state occurred during the Erligang period when it reached a supra regional level, defined by the construction of political centers requiring an estimated workforce thirty-five times larger than during the Erlitou [20]. This supra regional state was more stable than the previous states of the Longshan and Erlitou periods, and gave rise to both the Shang and Zhou dynasties and finally the first Empire of the Qin dynasty at the end of the last millennium BC.

But how much did this social complexification impact pig husbandry and the life of farming communities in China? Did the rise of a more complex society dictate new economic models that induced changes in pig production, or was the farming economy resilient to these socio-economic changes? In order to address these issues, we investigated the changes in the morphology and diet of pigs from two sites in the Henan Province: the rural village of XWG with 5,000 years of cultural changes from the Late Neolithic Yangshao culture (5000–3000 calibrated years Before Christ: i.e. cal. BC) up to the Han dynasty (206 cal. BC– 220 AD) [19]; and the XZ site, which was a flourishing fortified urban site whose occupation lasted from the Longshan period up to the rise of the Erlitou [22]. To track the changes in pig morphology and diet throughout the chronological sequence of XWG we combined dental geometric morphometrics and stable isotopic analyses. Dental form variation is used as a proxy of the divergence process between wild and domestic phenotypes, as well as being a marker of population history [10,23,24]. This morphological survey relied on a large scale comparative study including other Early and Middle Neolithic wild and domestic pigs from Northern, Central and Southern China. Stable carbon and nitrogen isotopes from the bone collagen of XWG pigs was used to trace the emergence of C_4_ foddering in pig husbandry practices, and an increased reliance on a high protein diet from domestic refuse. This is indicative of a change in husbandry practices from free-range to household reared, and a greater reliance on domestic and agricultural by-products [25–29].

## Materials and Methods

### Archaeological sites in Henan Province

#### Xiawanggang site (XWG)

**Fig 1:**
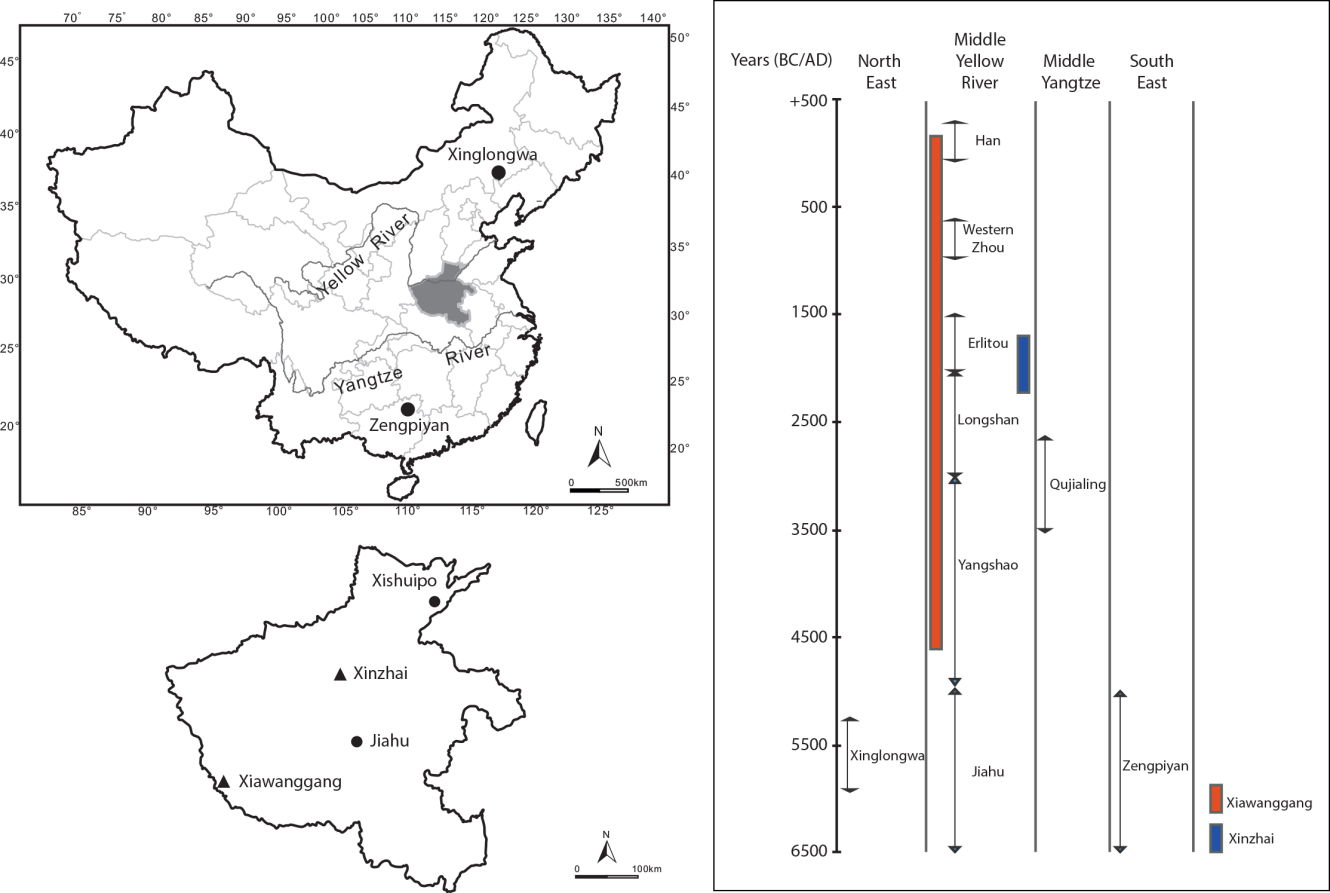
Location of the archaeological sites studied within their chrono-cultural context.

The site of XWG comprises a large rural village, covering at least 10,000 m^2^, with no archaeological evidence of a political elite. It is located on the Danjiang River in the extreme south of Henan Province (Fig 1). Although this site was previously excavated, from 1971 to 1974, all the animal bones analyzed in this study came from the more recent excavations of 2008 to 2010, under the direction of Professor He (IA-CASS). The site has a long chronological sequence of 5,000 years and a succession of cultural phases based on ceramic typological analysis: phase 1 (4600 cal. BC to 3400 cal. BC): the Middle Yangshao culture; phase 2 (3400 cal. BC to 2500 cal. BC): the Qujialing culture; phase 3 (2600 cal. BC to 1900 cal. BC): the Longshan culture; phase 4 (1850 cal. BC to 1550 cal. BC): the Erlitou culture; phase 5 (1060 to 770 cal. BC): the Western Zhou dynasty, and phase 6 (200BC to 200AD): the Han dynasty [30].

The natural environment is dominated by C_3_ plants. However, previous botanical analyses here, and on circum-adjacent sites, revealed that a millet and rice mixed agriculture existed from the Yangshao culture until, at least, the Western Zhou dynasty [31,32]. Such findings are supported by the stable isotope analyses of human and animal diets [33,34], which suggest that a complex agricultural system comprising multiple crop species had existed in that region since the Neolithic. No botanical analyses for the Han dynasty is available; although evidence that wheat occurred widely across the whole of China during that period prevails in ancient literature, such as *Nan du fu* 南都赋 (Zhang, 78–139 AD).

The farming economy was dominated by pig husbandry from the Yangshao up to the Han period. Overall, *Sus scrofa* remains make up the majority of the faunal assemblage, with more than 40% of the estimated Minimum Number of Individuals (MNI) (S1 Fig).

#### Xinzhai site (XZ)

XZ is located in Liuzhai County, Xinmi, Henan Province (Fig 1). It was first excavated in 1979; however, as with XWG, all the animal bone material studied came from the recent excavations of 2002 to 2004, directed by Professor Zhao (IA-CASS). XZ was a large urban settlement occupied from the Late Longshan period (ca. 2200 BC to 1900 BC) until the Early Erlitou culture (ca. 1750 BC). An elite presence on the site was inferred from the huge architectural foundations and the high quality of the artifacts (e.g. bronze vessel, jade, oracle bones). At its peak it covered up to 100 ha and was surrounded by a ditch and rammed earth wall [22]. So far, three phases have been identified according to ceramic analysis and ^14^C dating results: the first (2050 BC to 1900 BC), the second (1850 BC to 1750 BC) and the third (after 1750 BC) [22]. Due to its location, chronology and the richness of its archaeological features, the site of XZ is considered by some Chinese scholars to have been the capital of King Qi: the first emperor of the Xia dynasty [35,36].

In all three XZ phases, pigs are the main taxa comprising around 40% (MNI) of the faunal assemblage (S1 Fig).

### Geometric morphometrics (GMM)

We analyzed 344 specimens, of which 212 ancient and modern specimens (Table 1) were used as a comparative referential against the XWG (99) and XZ (33) specimens. Modern samples included the two wild boar sub-species of China [37]: *Sus scrofa ussuricus*, whose current range covers Northern China; *Sus scrofa moupinensis* in Southern China, and a sample of a Chinese domestic pig (unknown breed or location).

**Table 1:** Details of modern and archaeological specimens for GMM analysis. IZCAS: Institute of Zoology Chinese Academy of Sciences (Beijing, China); IVPP: Institute of Vertebrate Paleontology and Paleoanthropology (Beijing, China).

**Modern taxa**	**Status**	**Code**	**Curation**	**N**
*S*. *s*. *ussuricus*	Wild boar	WB_N	IZCAS	18
*S*. *s*. *moupinensis*	Wild boar	WB_S	IZCAS	5
*S*. *s*. *domestica*	Domestic	DOM	IVPP	6
**Archaeological site**	**Phase/Culture**	**Code**	**Date**	**N**
Xinzhai	3 /Erlitou	XZ3	1735-1705BC	4
	2 /Xinzhai	XZ2	1870-1750BC	15
	1 /Longshan	XZ1	2050-1900BC	14
Xiawanggang	6 /Han dynasty	XWG6	200BC-200AD	3
	5 /Western Zhou dynasty	XWG5	1060-770BC	27
	4 /Erlitou	XWG4	1850-1550BC	5
	3 /Longshan	XWG3	2600-1900BC	46
	2 /Qujialing	XWG2	3400-2500BC	7
	1 /Yangshao	XWG1	4600-3400BC	11
Xishuipo	Yangshao	XSP	5000-4000BC	113
Xinglongwa	2 /pre-Hongshan	XLW2	5700-5300BC	26
	1 /pre-Hongshan	XLW1	6100-5700BC	11
Jiahu	3 /Cishan_Peiligang	JH3	6200-5800BC	8
	2 /Cishan_Peiligang	JH2	6600-6200BC	6
	1 /Cishan_Peiligang	JH1	7000-6600BC	1
Zengpiyan	inland foragers	ZPY	8000-5000BC?	5

Comparative Neolithic samples included domestic pigs from Jiahu (JH; Cishan/Peiligang culture) and Xishuipo (XSP, Yangshao culture) in Henan Province [10] and wild boars from Xinglongwa (XLW), and Zengpiyan (ZPY) in Northern and Southern China [10].

The modern and archaeological Chinese specimens are all curated in a public state institution in China. The modern specimens came from two institutions, as indicated in Table 1. All the archaeological specimens are deposited in the Institute of Archaeology of the Chinese Academy of Social Science (IA CASS), 27 Wangfujing Street, Beijing, 100710, China, under the responsibility of one of the co-authors, Pr Yuan Jing. The museum reference numbers and the archaeological contexts for each specimen from this study can be found with the GMM dataset available at Figshare (doi: 10.6084/m9.figshare.3394915.).

#### Domestication and population changes through dental GMM

In order to track the domestic process and population changes in the archaeological record of the XWG and XZ sites, we used the second lower molar (M_2_) as a phenotypic marker to track population history [10,24], since its development shows less ecophenotypic plasticity than M_3._ Furthermore, M_2_ are measurable in wild and domestic pigs of between one and three years; while M_3_ is only be measurable in specimens over two years [38], which is another advantage to document domestication and husbandry processes.

The form of M_2_ was quantified with a geometric morphometric approach of its 2D occlusal view [10], combining landmarks on anatomical points located on the occlusal surface and semi-landmarks [39] equidistantly sampled along the external outline of the crown’s occlusal view.

All the forms’ configurations were standardized with a Procrustes superimposition to obtain comparable shapes cleared from scale, position and orientation. The semi-landmarks were slid on tangent lines to the crown’s outline using the bending energy sliding method [40]. The new set of shape (Procrustes coordinates) and size (centroid size) variables obtained after the Procrustes superimposition provided the input dataset for statistical analyses. The centroid size of the molar was computed as the square root of the sum of the squared distance of each landmark and semi-landmark, to the center of gravity of the points’ configuration.

Cartesian coordinates were digitized with TPS dig2.18 [41]. Procrustes superimposition and sliding method were performed with TPSrlw 1.59 [42].

#### GMM Statistics

The overall molar size difference among samples was tested with an Analysis of Variance (ANOVA), and graphically synthesized with a notched boxplot. The notch represents 95% interval confidence of the median, as it allows direct visualization of the size difference between two groups; overlapping notches would most likely mean that the two groups did not differ in size.

To identify the potential admixture of wild and domestic animals among the XWG and XZ samples, we relied on a statistical approach from the field of pattern recognition that explored data structuration without defining any prior grouping factors [43,44]. As we anticipated significant differences in molar size and shape among the wild boars and domestic pigs [10,45–47], we used a Bayesian model based clustering algorithm [48] applied to the M_2_’s centroid size and shape variations. Shape variables are represented by the first two principal components of the PCA performed on the variance covariance matrix computed from the Procrustes coordinates. This model provided the maximum likelihood estimations of the number of size clusters defined by the highest Bayesian information criteria (BIC).

The related change in the molar shape variation due to the size difference within the population samples, namely static allometry [49], was explored using a multivariate regression between the Log centroid size and the Procrustes coordinates, associated with a permutation test with 1000 runs. If the allometric effect was significant, it was corrected using the residuals of the pooled within group multivariate regression to obtain allometric free shape variables to improve the groups discrimination.

Molar shape comparison among groups was performed with a Multivariate Analysis of Variance (MANOVA) and the associated Wilk/Pillai test. Patterns of differentiation among groups were graphically displayed using a Canonical Variate Analysis (CVA): a multigroup Linear Discriminant Analysis (LDA) and a phenogram built with an unrooted Neighbour Joining (NJ) algorithm computed on Mahalanobis’ distances between the groups mean shapes. The shape changes along the discriminant axes of the CVA were produced by a multivariate regression approach [50].

The CVA and the NJ tree were performed after a dimensionality reduction of the shape variables using balanced samples to meet the discriminant analysis requirement using the mevolCVP function in R [51].

Statistical analyses were performed using MorphoJ version 1.02b (Copyright 2008–2013 Christian Peter Klingenberg) and R version 2.9.1. (R Development Core Team) with the libraries Rmorph [52], ape [53] and mclust [48].

### Stable isotope analysis

Stable carbon (δ^13^C) and nitrogen (δ^15^N) isotope ratios in animal bones primarily reflect those of the plants inherited along the food chain. Collagen δ^13^C values mainly reflect the protein part of the diet with a ^13^C-enrichment of 5‰ [54], while collagen δ^15^N values reflect diet and trophic levels with a stepwise ^15^N enrichment of 3–4‰ along the food chain [55,56]. Taking advantage of the stable carbon isotope signature of this C_4_ crop raised in environments dominated by C_3_ plants, this approach revealed an interwoven relationship between millet agriculture and animal husbandry in prehistoric China [57–59].

The scale of pig husbandry was easily assessed due to their reliance on the C_4_ food chain (cultivated millets and C_4_ fed domestic animals): higher δ^13^C and δ^15^N values were reflective of a greater contribution of agriculture and husbandry by-products. This may be indicative of different herding practices, from extensive to household herding, as well as differences in status (wild/domestic).

Assessment of the relative contribution of C_3_ and C_4_ plants to the pigs’ diet relied on the modern mean value of -26.5‰ for C_3_ plants from open areas (most of them ranged from -29 to -25‰). After correction for the fossil fuel effect (1.5‰; [60]) and consideration of a 5 ‰ ^13^C-enrichment between diet protein and collagen [61], it was estimated that a pure C_3_ diet would be reflected in bone collagen values ranging from -22.5‰ to -18.5‰ (averaging -20‰). By using the δ^13^C values [62] from archaeological millet seeds (-11.9 to -9.6‰) and modern millet leaves (including data for common and foxtail millets, both potentially present in Neolithic China; -14.6 to -12.3‰), a millet seeds-based diet would be expected to produce collagen δ^13^C values comprised of between -6.9‰ and -4.6‰, and a millet leaf-based diet of between -8.1‰ and -5.8‰. Using the cutoff value of -31.5‰ as indicative of the canopy effect in modern times [63], bone collagen δ^13^C values tending towards -25‰ or lower should reflect a contribution of C_3_ resources from the forest. These threshold values are approximations, and will be used to describe trends rather than to calculate the precise estimations of the relative contribution of C_3_/C_4_ plants to diet.

#### Selected samples of XWG pigs

After extracting the bone collagen (S1 Text) we selected 76 animal specimens and 5 humans from XWG (S1 Table). We targeted pigs with a fully erupted second molar, aged at least 8–10 months (dental stage 10–11 or older) [64], to avoid the influence of the suckling effect on the δ^15^N values [65]. The isotopic sampling strategy performed before the implementation of the GMM study prevented the matching of all isotopic signatures and dental form on the same specimen sample. However, 36 M_3_ linear measurements, but only 3 GMM values, could be matched with the δ^13^C and δ^15^N values (S2 Table).

Other taxa were sampled to contrast the isotopic signals of the pigs with species representative of the natural ecosystem:

- deer (*Cervus nippon*): these typical herbivores were expected to reflect the non- significant occurrence of C_4_ plants in the wild;
- badgers (*Meles leucurus*) and bears (*Selenarctos thibetanus*): these omnivores include animal protein in their diet which we would expect to see in wild boar and extensively raised pigs, although in suids this animal contribution would be significant only on a seasonal scale;
- tigers (*Panthera tigris*): these carnivores deliver δ^15^N values reflective of a high contribution of animal protein to diet.

#### Statistical study of the isotopic variables

To assess the clustering of the δ^13^C and δ^15^N values we used the same Bayesian model based clustering as previously described in the GMM section.

Differences in diet among all samples were compared for δ^13^C and δ^15^N values using ANOVA.

## Results

Exploratory analyses assessing the mixture of wild and domestic pigs in XWG and XZ clearly exclude the presence of wild boars or hybrids in the GMM dataset, and suggest that the domestic pigs of XWG had a greater size disparity than XZ (S2 Text).

### Molar size disparity in modern and ancient wild boars and pigs in China

**Fig 2:**
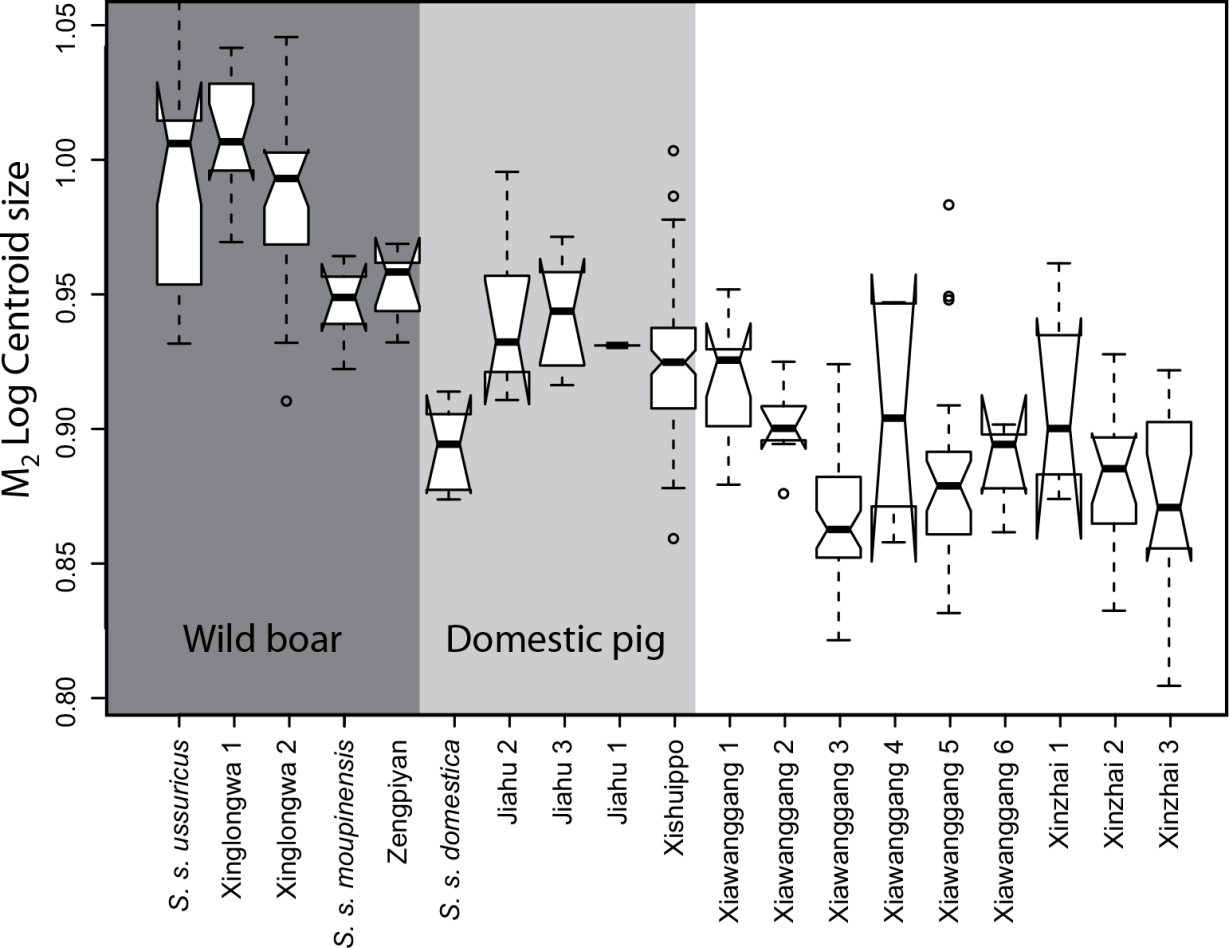
Notched boxplot of the M_2_ Log transformed centroid size for all samples. The box represents 50% of the variation around the median (horizontal black line); the brackets represent the minimum and maximum. Notches represent a 95% interval confidence of the median.

Molar centroid size variation in both current and past samples from China showed significant differences (*F* = 44.51, *p*<0.001). Modern and archaeological wild boars had the largest teeth: XLW samples were in the same range as *S*. *s*. *ussuricus*, and the ZPY samples were in the same range as *S*. *s*. *moupinensis* (Fig 2); which suggests that the size of wild boars in China has not changed during the last 10,000 years. Modern domestic pigs have smaller molars than the Early and Middle Neolithic pigs of JH and XSP. The JH pigs evidenced a molar size range overlapping with *S*. *s*. *moupinensis*.

The XWG samples, except for a minority of specimens in phases 1 and 4, were significantly smaller than the wild phenotypes of Northern and Southern China and displayed important molar size changes over time. The Yangshao specimens of phase 1 were in the same size range as the Yangshao pigs of XSP, but their size decreased significantly from phase 2 to reach a minimum during the Longshan period (phase 3). The size increased again from phase 4 and remained in the range of the modern domestic until phase 6. The XZ samples are in the size range of XWG and show a chronological trend of molar size decrease.

### M_2_ shape variation and the relationship between modern/ancient wild boars and pigs from China

**Fig 3:**
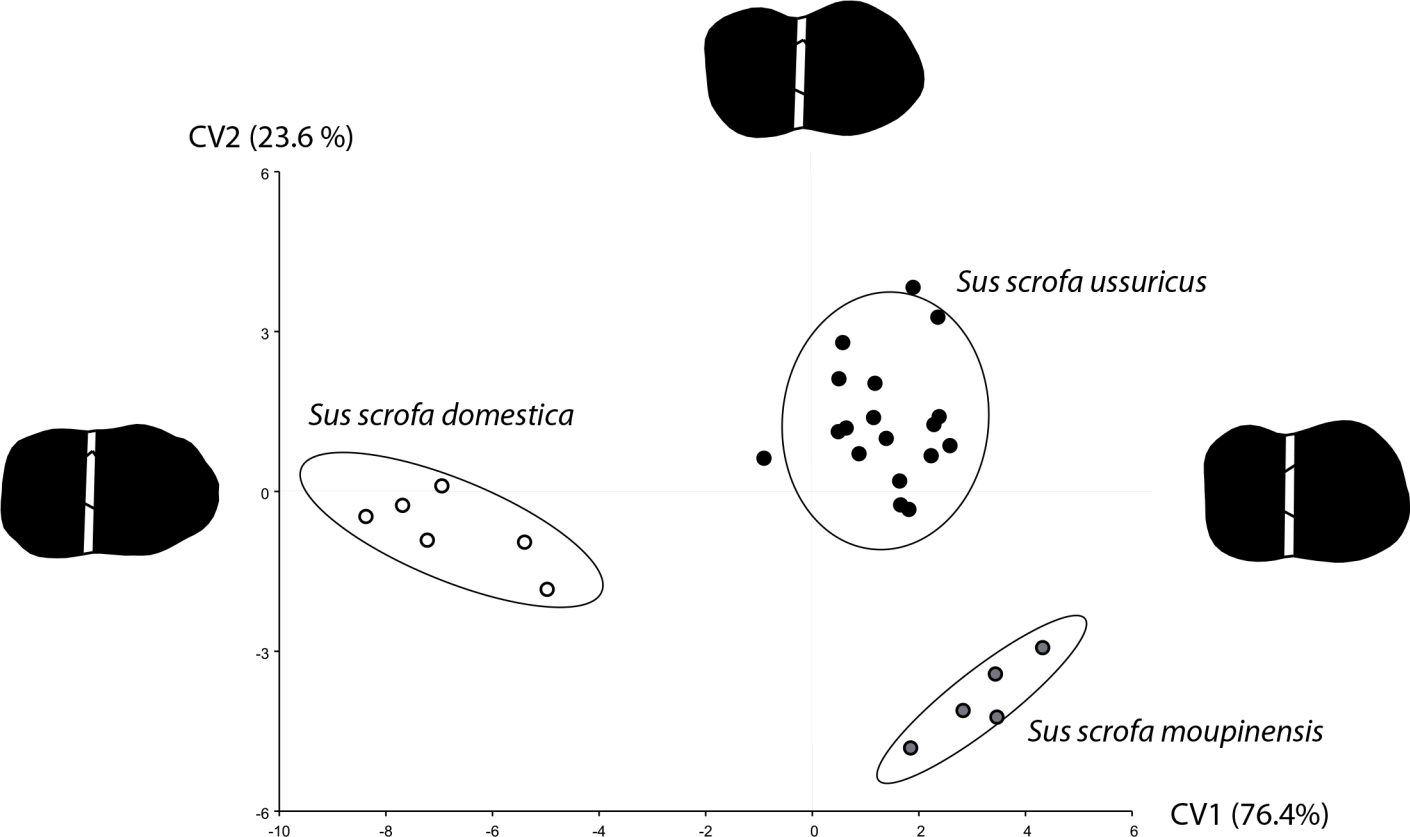
Molar shape differences between the two extent wild boar sub-species and the domestic pigs of China. First canonical variates (CV) computed on size corrected shape variables. The molar shape divergence between the wild and domestic type along the CV1 is displayed by shape reconstruction on each axes extremity; the divergence between the two wild boar sub-species is displayed along the CV2. Confidence ellipses contain 90% of the data points with a 0.9 probability.

Significant molar shape divergence (*P*<0.0001) exists among current wild and domestic *Sus scrofa* in China, characterized by a simplification of the molar shape with reduced cusps development on the labial and lingual sides (Fig 3).

Overall, we found significant differences in the modern/ancient wild and domestic *Sus scrofa* in China (*Pillai* = 1.68, *F* = 2.33, *Df* = 255, *den Df* = 4710, *P*<0.0001), cleared from a very small (less than 1%) but significant (*P* = 0.0317) allometric effect (see GMM Statistics).

**Fig 4:**
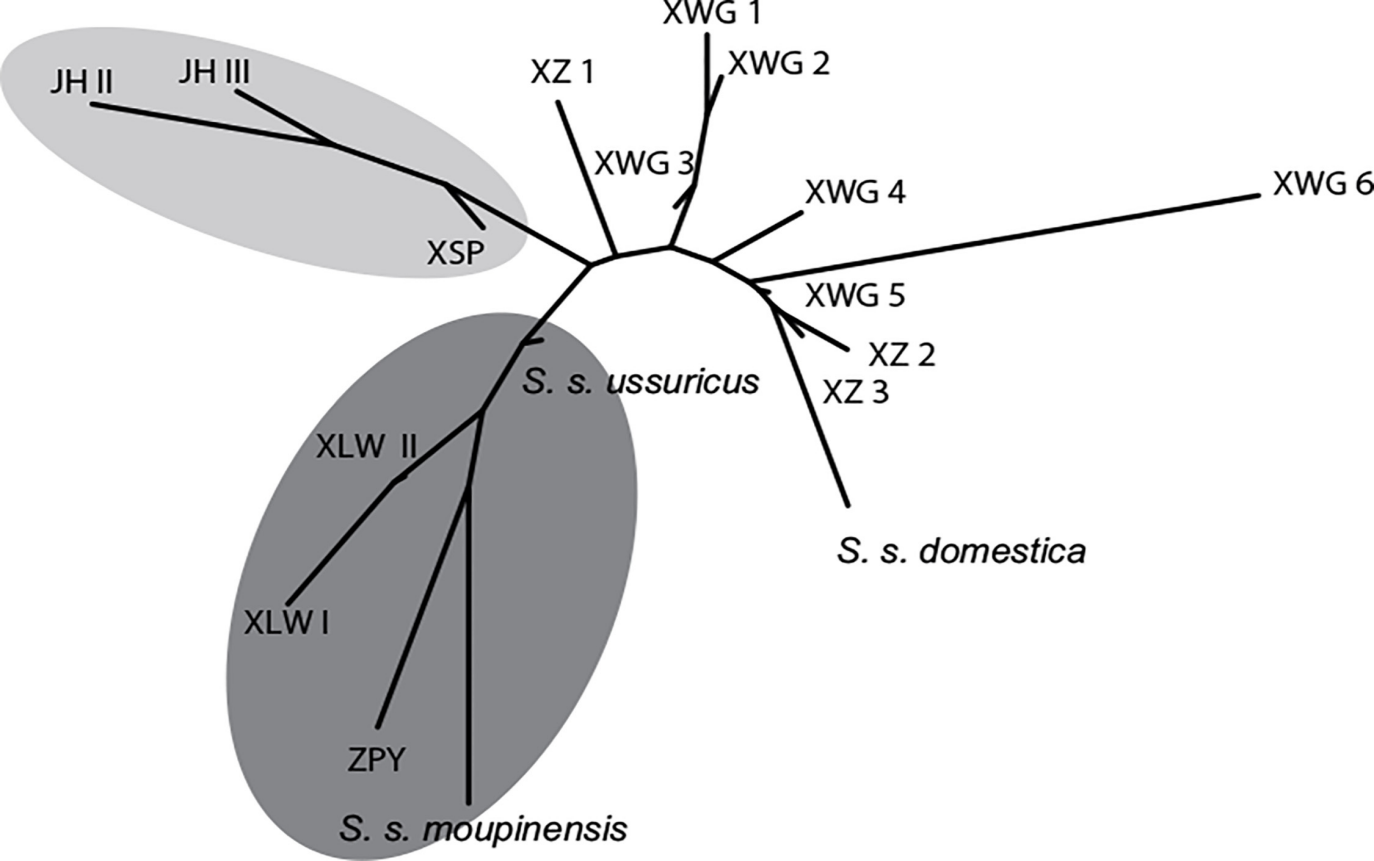
Dental shape relationships among modern and archaeological samples. Phenogram showing the M_2_ shape relationships between the geographic and chronological mean shape of modern and archaeological samples.

Molar shape relationships among modern/ancient Chinese *Sus scrofa* (Fig 4) are clustered along a continuum of differentiation, driven by the divergence between the modern wild boar from Southern China, at the bottom of the phenogram, and the modern domestic pigs in the top left. Within this continuum of phenotypic divergence three morphogroups were distinguishable: (1) the wild morphogroup, which included Southern and Northern Chinese wild boars from the Zengpiyan site and the specimens from XLW; (2) the early domestic morphotype with pigs of JH and XSP; and (3) the advanced domestic morphogroup which includes modern domestic pigs and the XZ and XWG samples.

#### Molar shape diversification over time at XWG and XZ

**Fig 5:**
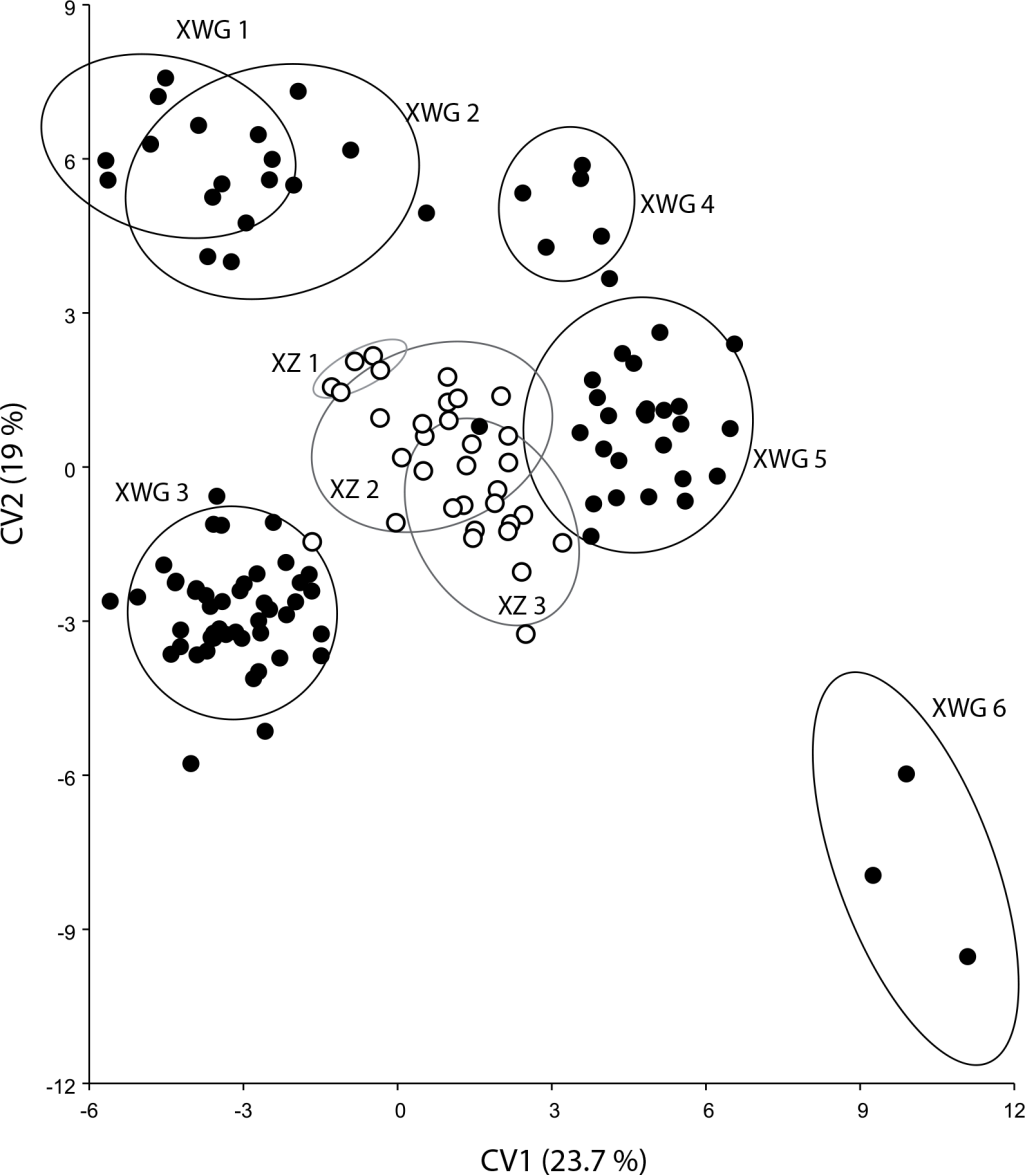
M_2_ shape differentiation among samples from XZ and XWG. First two axes of the CVA computed on size corrected shape variables. Confidence ellipses contain 90% of the data points with a 0.9 probability.

Most of the molar shape differentiation among the XWG and XZ chronological phases (Fig 5) was driven by a phenotypic trajectory along a temporal gradient. Only phase 3 from XWG departed from this chronological trajectory. Conversely, the XZ phases 1 to 3, contemporaneous with XWG phases 3 and 4, remained along this chronological trajectory; which suggests that pigs from phase 3 were either exogenous or have underwent severe developmental perturbations.

#### Distribution of the stable isotope values from animal bone collagen in Xiawanggang

**Fig 6:**
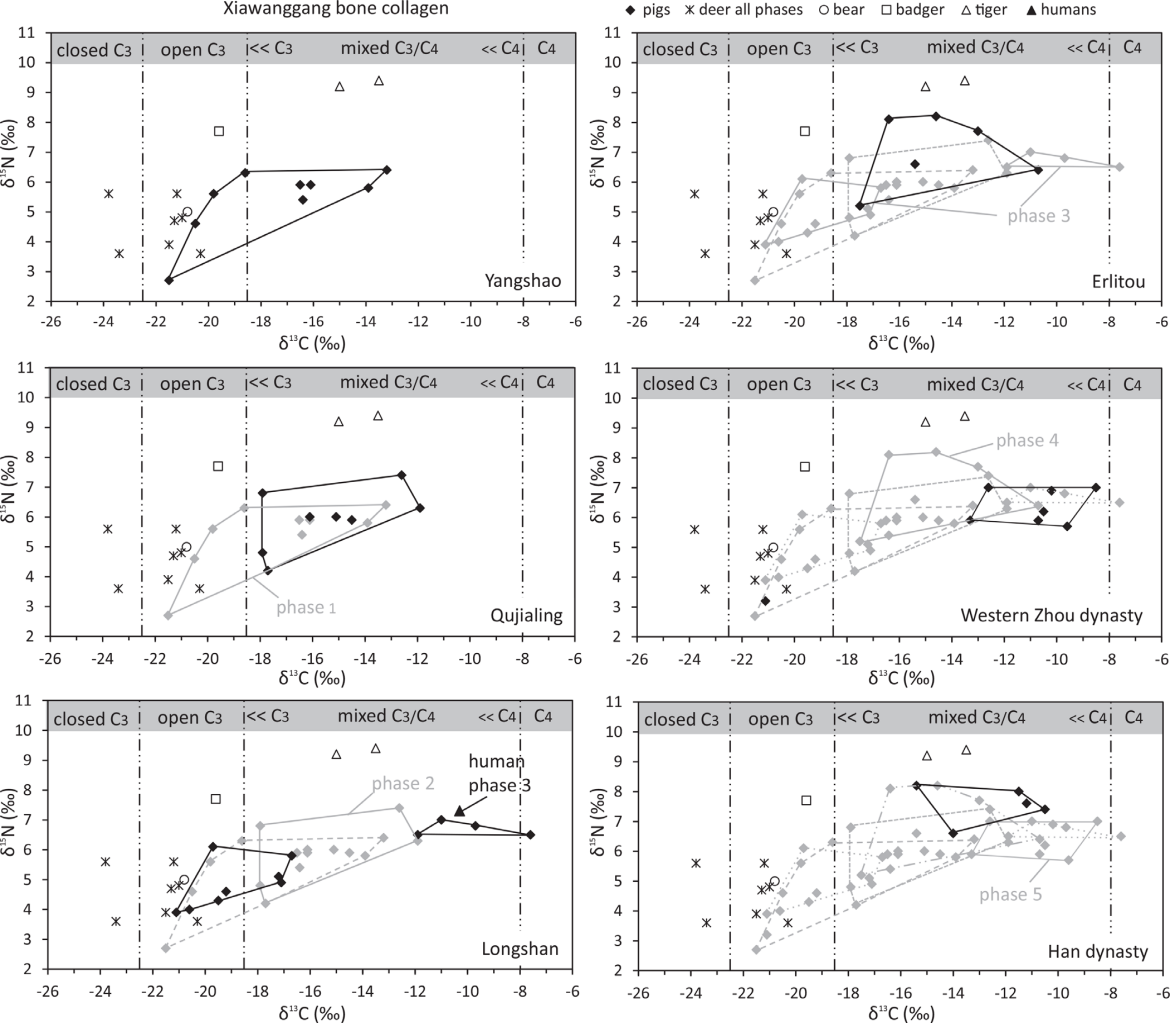
Scatter plot of the δ^13^C and δ^15^N values of bone collagen from the XWG site displayed per phase.

In total, seven deer from different phases had δ^13^C values ranging from -23.8 to -20.3 ‰ (average = -21.8 ‰), suggesting a pure C_3_ plant diet. Only two exhibited a contribution of resources from dense forest (S1 Table and Fig 6). The δ^13^C values measured in hares (-20.4 ‰), bears (-20.8 ‰) and badgers (-19.6 ‰) reflects a diet of C_3_ plants from open environments. These wild herbivores and omnivores had no access to C_4_ plants, which confirms their absence, or insignificance, in the surrounding wild environment of XWG.

In addition, the bone collagen δ^15^N values measured in the deers (3.6 to 5.6 ‰) and tigers (9.2 and 9.4 ‰) provided reference values for herbivores, and a super carnivore in the ecosystem of the XWG site. Unsurprisingly, the badgers delivered an intermediate value (7.7 ‰), corresponding to an omnivorous dietary behavior dominated by animal protein.

Significant differences were found across all XWG phases for both δ^13^C (ANOVA: *F* = 2.7, *p* = 0.03) and δ^15^N values (ANOVA: *F* = 4.1, *p* = 0.003): a chronological increase in C_4_ plant intake and the tropic levels of pigs from phases 1 to 6; a wide range of variation in both the δ^13^C (from -21.5‰ to -7.6‰) and δ^15^N values (from 2.7‰ to 8.2‰), encompassing pure C_3_ to pure C_4_ diets; and different trophic levels (Fig 6).

From the earliest occupations of the Neolithic (phase 1, Yangshao), millet contributed (either directly or indirectly) to the diet of the majority of pigs to varying degrees; although not as the main diet component, as some of the herds did not receive any (δ^13^C values lower than -18.5‰). The pigs’ δ^15^N values (5.4 ‰ on average) compared directly to those measured in deer, suggesting a dominantly herbivorous diet. In phase 2 (Qujialing), millet contributed to the diet of all the analyzed specimens with variable contributions among individuals, three of which were low (δ^13^C values around -18 ‰). The pigs’ trophic level was maintained at an herbivorous level, although slightly higher on average than in the previous phase (mean δ^15^N = 5.9 ‰).

However, a significant change was observed during the Longshan period (phase 3), where two groups of δ^13^C values were found and supported by Bayesian modelling (2 groups: log.likelihood = -44.25, n = 17, df = 4, BIC = -99.83). A group of eight specimens with δ^13^C values comprised between -21.1 and -16.7 ‰ evidenced a predominantly C_3_ diet, suggestive of either a limited contribution of millet or none at all. In contrast, a second group of four specimens with δ^13^C values comprised between -11.9 and -7.6 ‰ evidenced a heavy contribution of millet to diet (Fig 6). The C_4_ plant diet group also delivered higher δ^15^N values than the C_3_ plant diet group (mean δ^15^N values = 6.7 ‰, contra 4.8 ‰), suggesting a greater reliance on domestic refuse.

**Fig 7:**
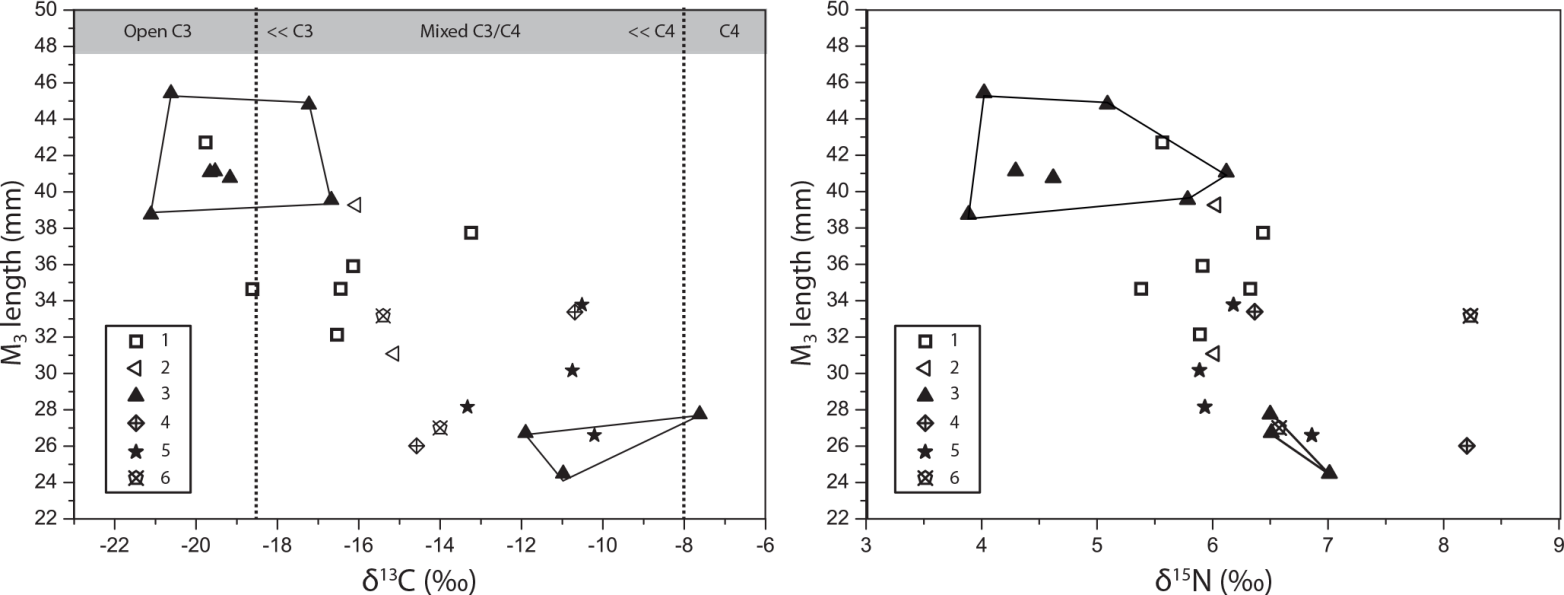
Relationship between ^13^C and ^15^N isotopic values and the M_3_ length measurements across XWG phases. Chronological phases of XWG are depicted by six different symbols. The Longshan specimens of phase 3 are clustered according to their M_3_ length, either below or above the 37 mm threshold.

Comparison of the M_3_ length measurements against the isotopic values (Fig 7) showed that specimens with a C_3_ predominant diet were “wild boar” sized (M_3_ length above the cut-off 37.9 mm [66]); while specimens with the C_4_ predominant diet and greater domestic refuse intake were of a much smaller “domestic” size (M_3_ length between 24.5 and 27.7 mm). This clearly suggests that both wild boars and domestic pigs were present in the Longshan phase assemblage of XWG. Wild boars were probably feeding in a C_3_ environment and occasionally raiding cultivated millet fields, which would explain the slightly higher δ^13^C values in three of them (-17.2 to -16.7 ‰), and small domestic pigs were feeding on millet foddering and domestic refuse. Since no M_2_ centroid sizes were in the range of current or Neolithic wild boars (Fig 2), the 46 small sized specimens from the GMM study most likely belong to the group of small pigs fed with a high intake of millet and domestic refuse. This is supported by the only matching specimen between the three analyses (S2 Table) whose M_2_ centroid size was related to a small M_3_ and a C_4_ diet.

Pigs from the Erlitou period (phase 4) showed a range of variation in δ^13^C values, lower than the Longshan period but similar to that measured in phase 2, suggesting a mixed C_3_/C_4_ plants diet with varying relative proportions of millet food. However, during this phase the pigs’ trophic level increased, with δ^15^N values in three individuals higher than those measured in badgers (Fig 7; mean δ^15^N = 7.0 ‰). The relative contribution of millet in most of the pigs’ diet increased during phase 5 (values comprised between -10.7 and -8.5 ‰), while δ^15^N values (6.0±1.2‰, n = 8) suggested a lower contribution of animal protein to diet compared to the previous phase. During the Han dynasty, the pigs’ δ^13^C values were slightly lower than those from the previous period; suggesting an increased consumption of wheat and rice (C_3_ crop) which was widely cultivated at the time, as attested to in the historical records (Zhang, 78–139 AD). A high trophic level was maintained throughout, as reflected in the highest δ^15^N values across the whole sequence of the site (mean δ^15^N = 7.6±0.6‰, n = 5).

Within each phase of occupation, inter-individual variability in the δ^13^C values measured in the pigs was high (from 6 ‰ to over 8 ‰ in phases 1, 2 and 4). Similarly, high inter-individual variability per phases was observed in the δ^15^N values (3.7 ‰ in phase 1, 3.1 ‰ in phase 2 and 3.0 ‰ in phase 4).

## Discussion

### Intensification of pig domestication in Late Yangshao XWG

Our survey of the diversification tempo in the *Sus scrofa* dental phenotype from a wild to a highly selected domestic breed, suggests an arrhythmic divergence over time with pulses of divergence instead of a gradual change. The wild phenotype encompasses the ancient wild boars of XLW and ZPY, and the two current wild boar sub-species: *S*. *s*. *ussuricus* and *S*. *s*. *moupinensis*. The first morphological divergence occurred between 8000 cal. BC in JH and, up to 4000 cal. BC in XSP, with an early domestic morphotype significantly divergent from the wild phenotype. This divergence is the result of a complex domestication process in which the phenotype of an animal (or small group of animals) is altered due to their forced separation from their wild relatives; and their subsequent adaptation through developmental and evolutionary processes, to the new genetic and environmental changes of their new anthropogenic habitat [67]. This morphological signature confirms the early domestication process from the beginning of the 7^th^ millennium BC in the middle Yellow River valley [10].

The amount of phenotypic changes, between the Early Yangshao pigs of XSP and the Late Yangshao pigs of XWG, suggests that the phenotypic diversification towards the current domestic phenotype experienced an acceleration observable from the Late Yangshao (from the mid-5^th^ millennium BC) onwards. This acceleration suggests a greater anthropogenic selective pressure, and/or a better control of wild genetic introgression [68]; the latter buffering the selection and divergence from the wild phenotype [69]. Whatever the process (i.e. higher selection or greater control of wild genetic introgression), they both indicate a greater investment in pig husbandry during the later phases of the Yangshao. This concurs with evidence for agriculture intensification from household archaeological remains [70], the ubiquity of large scale storage for millet and the abundance of improved agricultural tools [71,72] during the Middle and Late Yangshao. However, this substantial investment in pig husbandry, during the Yangshao phase of XWG, was not concomitant with a greater reliance on foddering from millet agriculture, as observed at other Yangshao sites [26,73]. Nevertheless, one cannot exclude that the low δ^13^C values in the Yangshao pigs could also have resulted in a greater reliance on rice (C_3_ crop) foddering, as suggested for the human isotopic signatures at JH [27] and the Gousan site [34].

#### Pig husbandry from the Yangshao to the Han dynasty in XWG: resilience and rupture

The common population history, evidenced from the dental phenotype, throughout 5,000 years of archaeological record in XWG, clearly suggests that pig farming relied on the same local livestock which gradually changed over time. Husbandry practices changed from mainly extensive herding during the Yangshao up to entirely household rearing during the Han dynasty, with a gentle increase of millet foddering and animal protein intake according to isotopic values. These gradual and related changes in the XWG pigs’ morphology and diet are characterized by the flexibility of its husbandry, and are observable across the entire XWG sequence through the high inter-individual variability in the pigs’ diet within each phase: specimens with no, low or 50% millet contribution. This could be related to inter-annual fluctuations in agricultural outputs, definitive of the level of redistribution of agricultural products between villagers and animals [58].

The only shift in this gradual change happened during the Longshan period. Here, we observed the occurrence of wild boars alongside a herd of very small and significantly different pigs, reared to an extent not seen until the Han dynasty and never during previous Neolithic phases. Their husbandry is characterized by a C_4_ dominated diet and a high animal protein intake; suggestive of household dependent management, where pigs were fed with foddering from millet agriculture by-products along with kitchen scraps [28].

But how can we explain this sudden occurrence of small, idiosyncratic and household fed pigs? Especially when we consider that pig farming continued to rely on the extensive husbandry of local livestock for millennia after this event. We considered three hypotheses that could provide plausible explanations for this rupture, involving climatic (first hypothesis) and politico-economic driving forces (second and third hypothesis).

The first hypothesis takes into account the cold and dry event of 4000 cal. BP, considered by many scholars to have led to the collapse of Neolithic societies [74]. Droughts would have caused drastic environmental stress that could have led to the collapse/decline of local pig herds, and the extensive herding practices of XWG, requiring the replacement of stock and a closer proximity of pig husbandry within the household. However, no clear evidence has been recorded for this climatic deterioration in East Asia outside the south-eastern coast of China [20]. Furthermore, such a collapse did not occur for the Late Longshan pig herds in neighboring XZ.

The two (antagonistic) politico-economic hypotheses consider that social complexification, during the Longshan period, triggered a new economic model driven by an emerging political power in order to gain staple wealth from domestic production [75,76]. The first, is a pro-elite hypothesis which considers that an emerging political power intensified pig production beyond the immediate needs of the community, in order to generate stable wealth and legitimize their power by promoting social inequality through feasting [75]. However, according to this pro-elite hypothesis, we would expect to see an increase in pig size during the Longshan period, along with an increase of their occurrence in burials (due to feasting) as a display of political power. On the contrary, our dataset shows a drastic decrease in pig size, and the archaeological evidence suggests a decline of pigs in burials during the Longshan period [77]. Indeed, during the Chinese Neolithic, the interment of pig skulls or of complete animals within human burials (as a display of social status differentiation) reached its peak during the Middle Neolithic, and then reduced during the Longshan, in both the Shandong [77] and the middle Yellow River [78,79]. This suggests that the Longshan elites did not invest in mortuary events to demonstrate their political and social power, but preferred to concentrate on the construction of community buildings and defensive walls which required greater public input [80]. Therefore, this pro-elite hypothesis is supported neither by our morphological dataset nor by the available archaeological record.

The second sociopolitical pathway is an anti-elite hypothesis which considers that pig production in a farming village, such as in XWG, could have been a way to avoid the incorporation of new economic models dictated by rising political authorities [81,82]. Household pig production, relying on domestic and farm refuse [83], would have provided farming communities with the flexibility to adapt to unfolding changes and/or evade enforced directives [84]. This anti-elite hypothesis would better suit our results for two main reasons. Considering the deleterious effect of confinement and poor diet on a suid’s body growth [85–87], a household management system could have induced developmental changes that would explain the drastic size reduction and morphological divergence from the free-ranging herd. This hypothesis would also explain why a size reduction, or a morphological divergence, was not observed at the elite site of XZ: the pigs consumed there (during the Longshan) were the larger free-range pigs, not the pigs from the household production of XWG.

### Conclusion

For the first time, we have considered the interactive dynamics between pig domestication and husbandry in the course of social complexification in ancient China, using a case study of the diachronic accumulation of pig remains from the Late Yangshao to the Han dynasty from XWG.

We found evidence for an intensification of pig domestication during the Late Yangshao in XWG; possibly promoted by a greater investment in the selective pressure of the pigs, and/or a greater prevention of genetic introgression from wild boars. We also observed a gradual transition, in local herds, from extensive to village husbandry during the social complexification of ancient China, suggesting a resilient pig farming economy, in XWG, in the face of socio-political change.

However, the Longshan period significantly disrupted this gradual change in pig husbandry, with the occurrence of unprecedented, small and idiosyncratic pigs fed exclusively from domestic and cultivation refuse. Among the climatic and politico- economic hypotheses we presented, our dataset seems to support the anti-elite hypothesis of XWG’s pig production being used as a barrier against the new economic model of intensive domestic production driven by the Longshan elite.

However, our discussion must be viewed as exploratory. Further multidisciplinary studies covering as much cultural and sociopolitical entities as possible, are required to fully understand the role played by pig husbandry in the making of China’s complex societies.

## Supporting Information

S1 Fig **Faunal Spectrum of Xiawanggang (A) and Xinzhai (B).** Relative proportion of the taxa occurring in zooarchaeological remains counted according to the Number of Individual Specimens (NISP) or the Minimum Number of Individuals (MNI).
(EPS)

S1 Table Sample information and isotope data of the animal bones from the Xiawanggang site. (XLSX)

S2 Table Pigs samples from the Xiawanggang site with both isotopic and morphometric data. (XLSX)

S1 Text Methodological details of the bone collagen extraction for the isotopic studies. (DOC)

S2 Text Results of the preliminary study of size and shape variation for XWG and ZX specimens to assess the potential mixture of wild and domestic pigs for each phase using a Bayesian Model based clustering. (DOCX)
