# Correction: Social Complexification and Pig (*Sus scrofa*) Husbandry in Ancient China: A Combined Geometric Morphometric and Isotopic Approach

**DOI:** 10.1371/journal.pone.0162134

**Published:** 2016-08-25

**Authors:** The PLOS ONE Staff

There is a shading error in the three molar shape reconstructions in Fig 3. Please view Fig 3 here. The publisher apologizes for the error.

**Fig 3:**
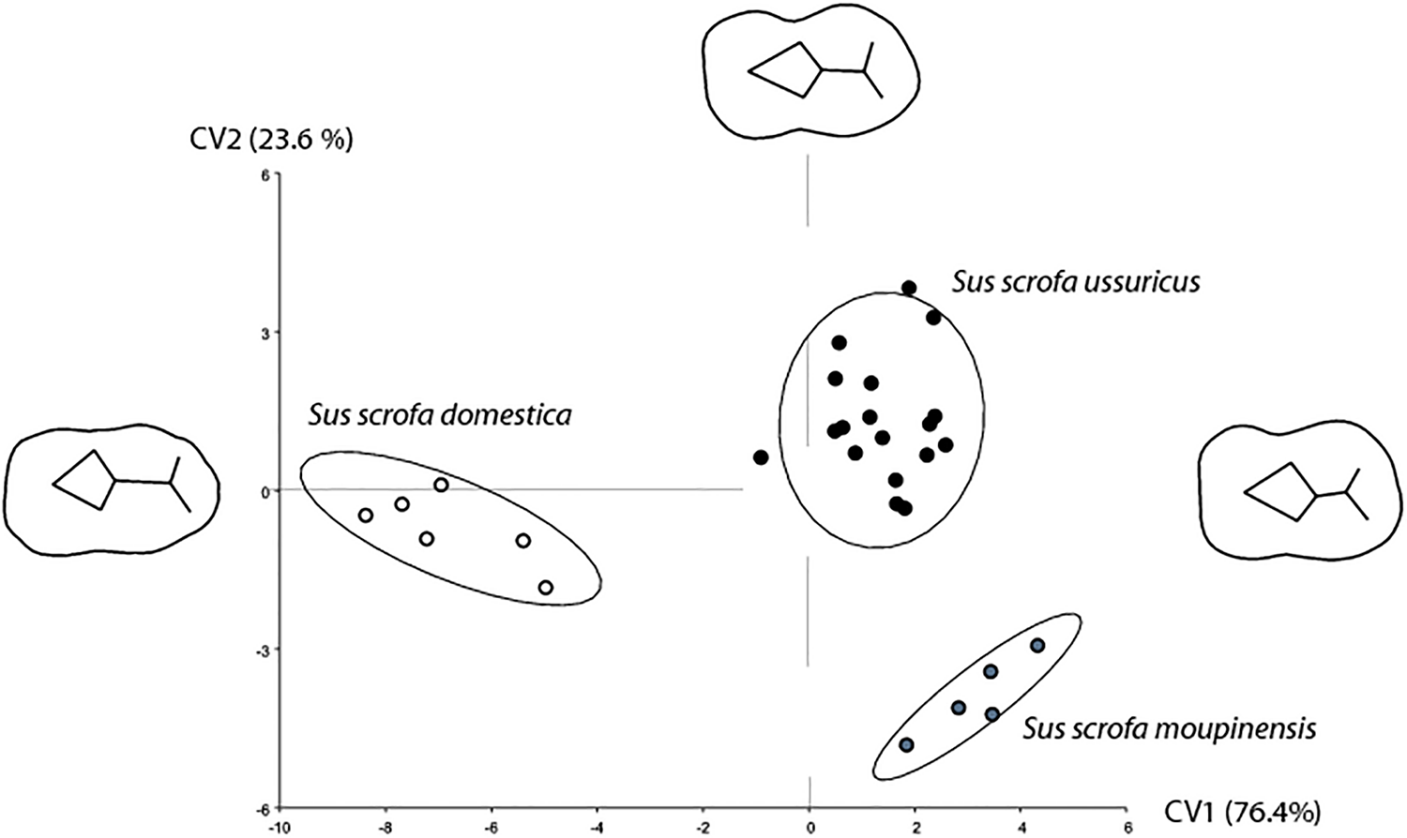
Molar shape differences between the two extent wild boar sub-species and the domestic pigs of China. First canonical variates (CV) computed on size corrected shape variables. The molar shape divergence between the wild and domestic type along the CV1 is displayed by shape reconstruction on each axes extremity; the divergence between the two wild boar sub-species is displayed along the CV2. Confidence ellipses contain 90% of the data points with a 0.9 probability.
