# Supplementary material for: Social Complexification and Pig (Sus scrofa) Husbandry in Ancient China: A Combined Geometric Morphometric and Isotopic Approach
Source: PLoS One. 2016 Jul 6;11(7):e0158523. doi: 10.1371/journal.pone.0158523 (PMC4934769; doi:10.1371/journal.pone.0158523)
Supplement: S1 Text — (DOC) [file pone.0158523.s004.doc]

**Bone collagen extraction**

Bone collagen was extracted from 200~300 mg of bone powder following (50). C and N contents and δ^13^C and δ^15^N values were measured on an Elementar Vario elemental analyzer interfaced to an Isoprime 100 IRMS. The standard for measuring the carbon and nitrogen content was Sulfanilamide. IEAE-N-1 and USGS 24 were used as standards to normalize N_2_ (AIR as standard) and CO_2_ (VPDB as standard) respectively. A collagen laboratory made standard with an average δ^13^C value of -14.7‰, and an average δ^15^N value of 6.8‰ was inserted for calibration. The analytical precision for δ^13^C and δ^15^N values was 0.2‰. Collagen were extracted from 61 out of 81 samples with very low yield (~56mg/g in average contra approximately 200 mg/g in fresh bone), indicating that the majority of bone collagen had decomposed during the long term burial. However, the assessment on key criteria of C (15.3%~47%), N (5.5%~17.3%) contents and the C/N ratios (2.9 ~ 3.6) (51,52) suggest that 60 samples were reliable for stable isotopic analysis.
