# Supplementary material for: Social Complexification and Pig (Sus scrofa) Husbandry in Ancient China: A Combined Geometric Morphometric and Isotopic Approach
Source: PLoS One. 2016 Jul 6;11(7):e0158523. doi: 10.1371/journal.pone.0158523 (PMC4934769; doi:10.1371/journal.pone.0158523)
Supplement: S2 Text — (DOCX) [file pone.0158523.s005.docx]

Mixture analyses found two most likely groups of molar size (likelihood=125.59, BIC=232.80) in all but the last XWG phases (Fig 1A, Table 1) but only one most likely group (likelihood=499.727, BIC=985.66) of molar shape. For phases 1 to 5, the small sized specimens are dominant, except for phase 1 where larger individuals are dominant. Such size bimodality has not been found in XZ (Fig 3B).

In order to check whether the large sized specimens of phases 1 to 5 were related to wild or hybrid phenotypes, their relationship within the wild and domestic dental shape dichotomy has been explored splitting each XWG phases in a A (small) and B (large) groups according to the Table 1. The phenogram (Figure 2) show that groups A and B cluster within the advanced domestic morphogroup but display longer branches due to small sample size. Since none of the large sized groups from XWG related either with the intermediate domestic group or with the wild morphogroup, we can conclude that the M_2_ size bimodality in the XWG samples is most likely due to the co-occurrence of small and large domestic morphogroups, rather than the presence of wild boars or hybrids. For these reason we have pooled them for the analysis as domestic specimens.


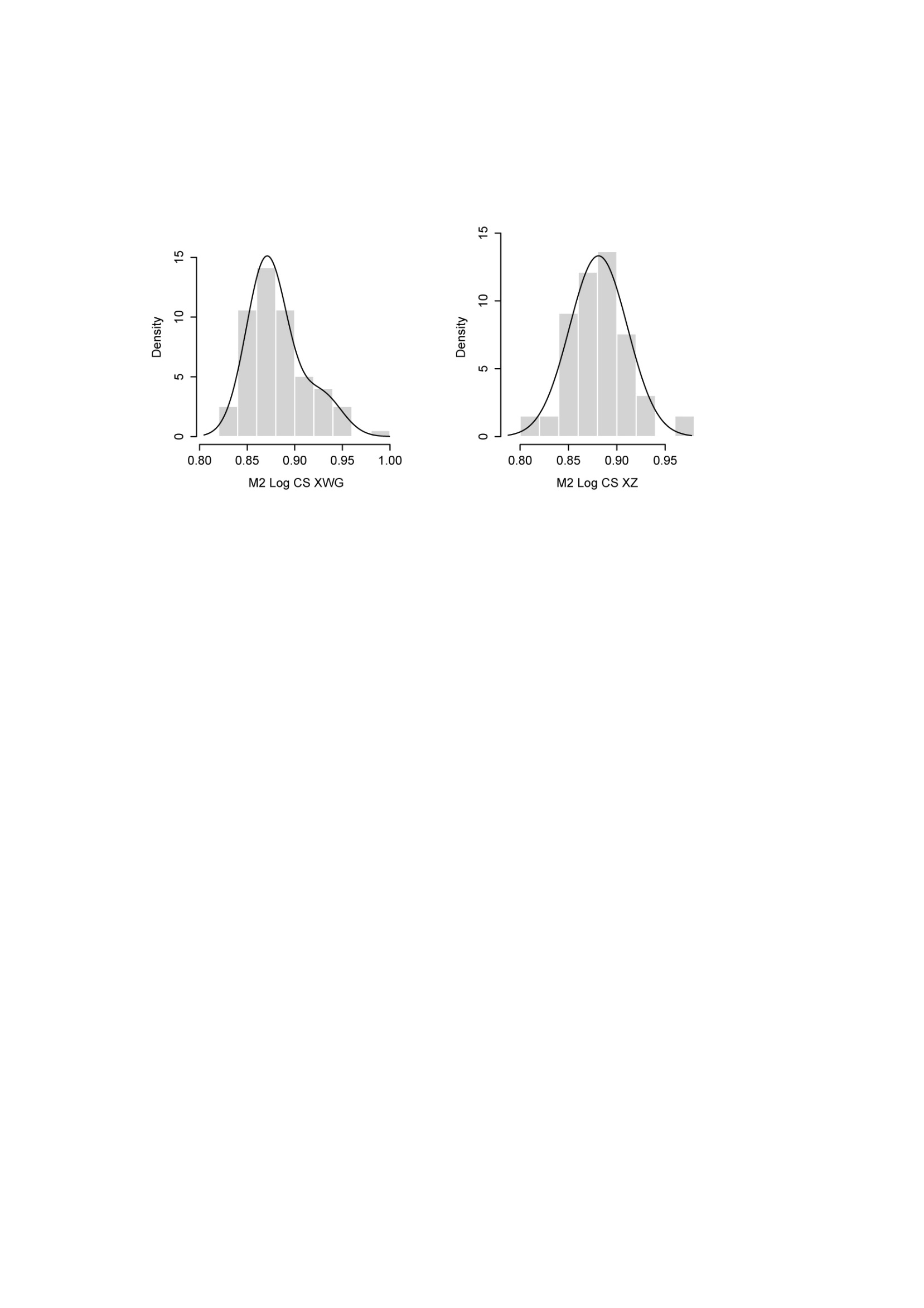


**Figure 1. Kernel density after Model-based clustering of the log-centroid size dataset from XWG (left) and XZ (right).**

|  | XWG phase 1 | XWG phase 2 | XWG phase 3 | XWG phase 4 | XWG phase 5 | XWG phase 6 |
| --- | --- | --- | --- | --- | --- | --- |
| Small | 4 | **5** | **43** | **3** | **24** | **3** |
| Large | **7** | 2 | 3 | 2 | 3 | 0 |

**Table 1: Number of M_2_ classified as small and large per chronological phase according to the Model-based clustering.**


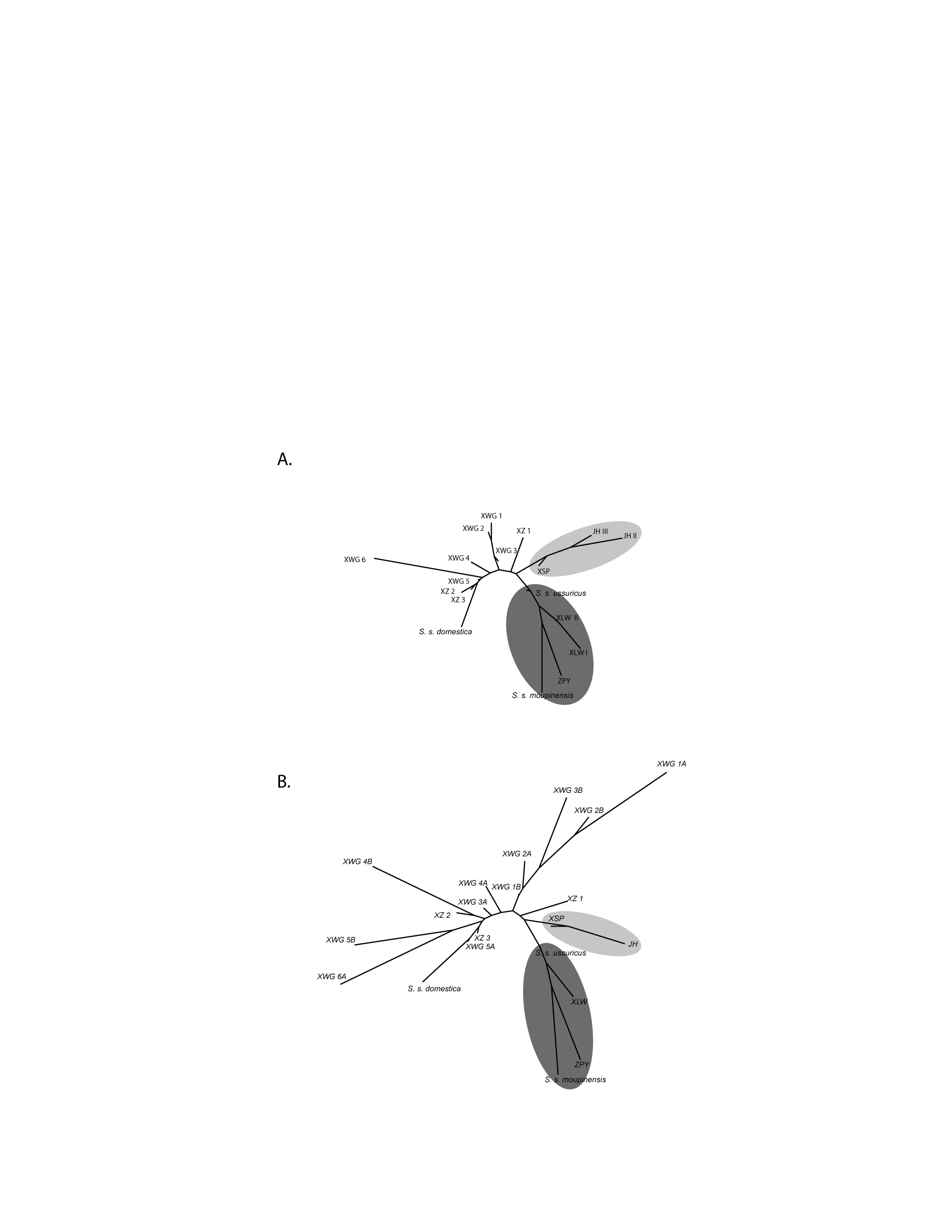


**Figure 2. Dental shape relationships among modern and archaeological samples.** Phenogram showing the M_2_ shape relationships between geographic and chronological mean shape of modern and archaeological samples with XWG phases separated into two groups of size (A: small, B: large) according to the Bayesian modelling classification.
